# Oxygen saturation targets for adults with acute hypoxemia in low and lower-middle income countries: a scoping review with analysis of contextual factors

**DOI:** 10.3389/fmed.2023.1148334

**Published:** 2023-04-17

**Authors:** Austin Herbst, Swati Goel, Abi Beane, B. Jason Brotherton, Dingase Dula, E. Wesley Ely, Stephen B. Gordon, Rashan Haniffa, Bethany Hedt-Gauthier, Felix Limbani, Michael S. Lipnick, Samuel Lyon, Carolyne Njoki, Peter Oduor, George Otieno, Luigi Pisani, Jamie Rylance, Mark G. Shrime, Doris Lorette Uwamahoro, Sky Vanderburg, Wangari Waweru-Siika, Theogene Twagirumugabe, Elisabeth Riviello

**Affiliations:** ^1^Department of Medicine, Beth Israel Deaconess Medical Center, Harvard Medical School, Boston, MA, United States; ^2^Department of Medicine, Perelman School of Medicine at the University of Pennsylvania, Philadelphia, PA, United States; ^3^Centre for Inflammation Research, University of Edinburgh, Edinburgh, United Kingdom; ^4^Network for Improving Critical Care Systems and Training, Colombo, Sri Lanka; ^5^Nat Intensive Care Surveillance-MORU, Colombo, Sri Lanka; ^6^Kijabe Hospital, Kijabe, Kenya; ^7^Clinical Research, Investigation, and Systems Modeling of Acute Illness Center, University of Pittsburgh, Pittsburgh, PA, United States; ^8^Malawi-Liverpool-Wellcome Trust Clinical Research Programme, Blantyre, Malawi; ^9^Division of Allergy, Pulmonary, and Critical Care Medicine, Vanderbilt University Medical Center, Nashville, TN, United States; ^10^Critical Illness, Brain Dysfunction, and Survivorship Center, Vanderbilt University Medical Center, Nashville, TN, United States; ^11^Geriatric Research, Education, and Clinical Center, Tennessee Valley Healthcare System, Nashville, TN, United States; ^12^Liverpool School of Tropical Medicine, Liverpool, United Kingdom; ^13^University College London Hospitals, London, United Kingdom; ^14^University Hospital-Kotelawala Defence University, Boralesgamuwa, Sri Lanka; ^15^Department of Global Health and Social Medicine, Harvard Medical School, Boston, MA, United States; ^16^Hypoxia Research Laboratory, University of California, San Francisco, San Francisco, CA, United States; ^17^Center for Health Equity in Surgery and Anesthesia, University of California, San Francisco, San Francisco, CA, United States; ^18^Department of Anesthesia and Perioperative Care, University of California, San Francisco, San Francisco, CA, United States; ^19^Harvard Medical School, Boston, MA, United States; ^20^Department of Surgery, Faculty of Health Sciences, Egerton University, Nakuru, Kenya; ^21^Mahidol Oxford Tropical Medicine Research Unit, Bangkok, Thailand; ^22^Mercy Ships, Lindale, TX, United States; ^23^College of Medicine and Health Sciences, University of Rwanda, Kigali, Rwanda; ^24^University Teaching Hospital of Kigali, Kigali, Rwanda; ^25^Division of Pulmonary, Critical Care, Allergy, and Sleep Medicine, Department of Medicine, University of California, San Francisco, San Francisco, CA, United States; ^26^Department of Anaesthesia, Aga Khan University, Nairobi, Kenya; ^27^University Teaching Hospital of Butare, Butare, Rwanda; ^28^Division of Pulmonary, Critical Care, and Sleep Medicine, Department of Medicine, Beth Israel Deaconess Medical Center, Harvard Medical School, Boston, MA, United States

**Keywords:** LMICs, Africa, context, oxygen saturation targets, SpO_2_

## Abstract

Knowing the target oxygen saturation (SpO_2_) range that results in the best outcomes for acutely hypoxemic adults is important for clinical care, training, and research in low-income and lower-middle income countries (collectively LMICs). The evidence we have for SpO_2_ targets emanates from high-income countries (HICs), and therefore may miss important contextual factors for LMIC settings. Furthermore, the evidence from HICs is mixed, amplifying the importance of specific circumstances. For this literature review and analysis, we considered SpO_2_ targets used in previous trials, international and national society guidelines, and direct trial evidence comparing outcomes using different SpO_2_ ranges (all from HICs). We also considered contextual factors, including emerging data on pulse oximetry performance in different skin pigmentation ranges, the risk of depleting oxygen resources in LMIC settings, the lack of access to arterial blood gases that necessitates consideration of the subpopulation of hypoxemic patients who are also hypercapnic, and the impact of altitude on median SpO_2_ values. This process of integrating prior study protocols, society guidelines, available evidence, and contextual factors is potentially useful for the development of other clinical guidelines for LMIC settings. We suggest that a goal SpO_2_ range of 90-94% is reasonable, using high-performing pulse oximeters. Answering context-specific research questions, such as an optimal SpO_2_ target range in LMIC contexts, is critical for advancing equity in clinical outcomes globally.

## Introduction

Guidelines for best-practice oxygen saturation (SpO_2_) targets in acutely hypoxemic adults are based entirely on evidence from high-income countries (HICs), which encompass only 16% of the world’s population (1–11). This can be problematic for clinical staff, clinical teachers, and researchers in low-income and lower-middle income countries (collectively LMICs) who are working to improve patient outcomes with oxygen therapy. As Chowdhury et al. note when examining a case of stroke care in an LMIC setting, “evidence-based standards of care cannot be separated from the contexts in which they are produced” (12). This is not simply a need to acknowledge that certain recommended interventions may not be available in all settings. Features of a given context may actually shift the risk-benefit balance of an intervention toward a different best-practice standard of care; differences in context could mean that a given intervention that leads to better outcomes in one setting could lead to neutral or worse outcomes in another. There is a specific example of this complexity in the sepsis literature, where fluid resuscitation volumes found to be life-saving or neutral in multiple HICs were found to be harmful in LMICs (13–18). The reasons for this may have to do with differences in the underlying etiologies of sepsis, timing of patient presentation, and lack of availability of ventilators to “rescue” patients from fluid overload (19).

Not only does all of the evidence on optimal SpO_2_ target ranges emanate from HICs, but also the evidence does not definitively point to an optimal SpO_2_ range (2–11, 20). Our research team is planning a trial of high flow versus standard flow oxygen delivery in five hospitals in three LMIC countries in sub-Saharan Africa (Kenya, Malawi, and Rwanda) (21). To ensure consistent practices in the two arms of the trial, we need to choose an SpO_2_ range to target for titrating oxygen therapy in both arms. The trial itself is based on the premise that context matters: the question of whether high flow oxygen is superior to standard flow has been explored in multiple HIC settings (22), but different epidemiology and resources may change the answer as to whether high flow oxygen should be used in LMIC settings for the best patient- and systems-level outcomes.

## Methods

To identify the optimal SpO_2_ range for patients enrolled in the trial, we conducted a scoping literature review regarding SpO_2_ targets for patients with hypoxemia (23). We reviewed prior interventional trials in hypoxemic adults to determine the precedent of SpO_2_ target ranges set in research protocols. We examined SpO_2_ target guidelines from national and international respiratory and critical care organizations for patients with hypoxemia. We also looked at direct evidence from trials comparing patient outcomes using different SpO_2_ target ranges. We explored literature regarding context-specific considerations that could impact the choice of best-practice SpO_2_ range in LMICs, including the relative inaccuracy of pulse oximetry in patients with darker skin pigmentation and shock, the need to conserve oxygen resources for all patients (24, 25), the lack of consistent access to arterial blood gases, which necessitates consideration of hypercapnic patients, and the impact of altitude on SpO_2_ (26–28).

### SpO_2_ ranges used in previous interventional trials

In previous ARDSNet trials evaluating mortality outcomes with interventions in patients with acute respiratory distress syndrome (ARDS), target oxygenation has been set at 88–95% (Table 1). The landmark trial of low tidal volume ventilation in ARDS chose this target range for study participants (29). The average level of arterial partial pressure of oxygen (PaO_2_; SpO_2_ not reported) reflects this and was similar between both groups when measured at days 1, 3, and 7 (range 73–77 mmHg). More recent studies assessing proning and paralysis in ARDS used this same 88–95% range without differences in measured PaO_2_ between groups (30, 31). These studies were all conducted in patients in HICs admitted to intensive care units with ARDS.

**TABLE 1:** Oxygen targets used in prior trials enrolling hypoxemic adults.

Trial	Target SpO_2_	Population
ARDSNet Low TV (29)	88–95%	Intubated ARDS patients
PROSEVA (30)	88–95%	Intubated ARDS patients
PETAL (31)	88–95%	Intubated ARDS patients
FLORALI HFNC (32)	≥92%	Non-intubated patients with acute hypoxemic respiratory failure with P:F ratio ≤300
HIGH RCT (33)	≥95%	Non-intubated immunocompromised patients with acute hypoxemic respiratory failure
Recovery-RS (34)	“Across all groups, local policies and clinical discretion informed decisions”	Non-intubated COVID-19 patients with acute hypoxemic respiratory failure

SpO_2_, oxygen saturation; ARDS, acute respiratory distress syndrome; P:F, ratio of partial pressure of arterial oxygen to the fraction of inspired oxygen; HFNC, high flow nasal cannula; RCT, randomized control trial.

Other studies that assessed acute hypoxemic respiratory failure (AHRF) in non-intubated patients have used a higher SpO_2_ cutoff (Table 1). The FLORALI trial of high flow oxygen, non-invasive ventilation, and standard flow oxygen in acutely hypoxemic adults used a target SpO_2_ of ≥92%; the HIGH trial of high flow versus standard oxygen in immunocompromised adults with acute hypoxemia targeted ≥95% (32, 33). The Recovery-RS trial, which was conducted to assess optimal non-invasive respiratory support modalities in patients with COVID-19 and AHRF, did not set a specific oxygen saturation target and instead deferred to individual study site policies and clinical discretion (34).

### Society guidelines for SpO_2_ targets

Multiple professional societies provide recommendations and guidance for SpO_2_ targets for patients with AHRF (Table 2). The British Thoracic Society (BTS) guidelines recommend a higher oxygenation goal of 94–98%, except for patients at risk for hypercapnia, for which they recommend a goal of 88–92% (35). Other groups, including the World Health Organization (WHO), Society of Critical Care Medicine (SCCM) Surviving Sepsis campaign, and the Thoracic Society of Australia and New Zealand, advocate for a more conservative oxygenation goal typically of at least ≥90%, except again for patients at risk for hypercapnia, where they recommend 88–92% (36–38). In 2018, a British Medical Journal (BMJ) Expert panel evaluated data on oxygenation targets in multiple clinical scenarios, including respiratory failure, myocardial infarction, and acute stroke. The expert panel recommended a general oxygenation goal of 90–94% for most patients, with an adjusted goal of 88–92% for those at risk of hypercapnia (39). The American Thoracic Society (ATS) does not specifically cite an oxygenation target in their guidelines, with the exception of 88–92% for non-invasive ventilation for patients at risk of hypercapnia (40, 41).

**TABLE 2:** Society guidelines for oxygen saturation targets.

Society/Guidelines	Recommended target	Level of recommendation
British Thoracic Society (BTS) (35)	94–98% for most patients 88–92% for those at risk of hypercapnia	–Grade D Recommendation –Grade A for COPD, Grade D for other conditions
World Health Organization (WHO) COVID-19 guidelines (37)	≥94% for those with emergency signs during resuscitation >90% once patients are stable	–COVID-19 specific recommendations
Society of Critical Care Medicine (SCCM) Surviving sepsis campaign, COVID-19 recommendations (36)	90–96%	–Strong recommendation
Thoracic Society of Australia and New Zealand (38)	92–96% for most patients 88–92% for risk of hypercapnia	–Grade B Recommendation
British Medical Journal (BMJ) Expert panel (39)	90–94% for most patients 88–92% for risk of hypercapnia	– Recommended not starting supplemental oxygen on patients with acute MI or CVA with SpO_2_ ≥ 90% (if SpO_2_ 90–92%, weak recommendation; if SpO_2_ ≥ 93%, strong recommendation)
American Thoracic Society (ATS) (40, 41)	No specific oxygenation targets specifically commented on in ATS guidelines for general hypoxemic patients 88–92% for non-invasive ventilation for patients at risk of hypercapnia	

### Direct evidence for different SpO_2_ target ranges

An understanding of the potential risks of both hypoxemia and hyperoxia has driven investigations of the optimal SpO_2_ target range for different populations of patients. While we know that hypoxemia can result in tissue ischemia and death, we also know that hyperoxia can cause oxidative stress and inflammation, with the potential for negative clinical consequences (42–44). In critically ill (not necessarily hypoxemic) patients, a prior practice of allowing or even targeting hyperoxia to promote tissue oxygenation has been found to be harmful (42, 45). In acutely hypoxemic patients, the question is more complicated, as potentially harmful levels of FiO_2_ may be needed to reach “normoxia” in some patients; this raises the question of whether not only an avoidance of hyperoxia, but even pursuing some level of permissive hypoxemia, could be beneficial (43).

Many recent studies have assessed conservative versus liberal SpO_2_ goals in different patient populations, with the weight of evidence suggesting that lower (“conservative”) SpO_2_ targets are not harmful and may even produce superior outcomes as compared with higher (“liberal”) SpO_2_ targets in some cases. Table 3 outlines these studies, patient populations, and their specific comparisons, all of which occurred in HICs.

**TABLE 3:** Studies of conservative versus liberal oxygen saturation goals.

References	Study design	Conservative [# patients]		Liberal [# patients]		Outcome
**Studies of patients with acute hypoxemic respiratory failure and ventilated patients**						
Panwar et al. (5)	RCT in mechanically ventilated patients	SpO_2_ 88–92% [52]		SpO_2_ ≥ 96% [51]		–No significant difference in organ dysfunction, ICU mortality, or 90-day mortality
ICU-ROX (2)	RCT in mechanically ventilated ICU patients	SpO_2_ 90–97%, FiO_2_ decreased to 0.21 if SpO_2_ > 90% [484]		SpO_2_ with no specific upper limit, > 90% [481]		–No significant difference in ventilator free days No significant difference in 180 day mortality
LOCO_2_ (10)	RCT in patients with ARDS	PaO_2_ 55–70 mmHg (SpO_2_ 88–92%) [99]		PaO_2_ 90–105 mmHg (SpO_2_ ≥ 96%) [102]		–Prematurely stopped due to safety concerns and low likelihood of significant difference –34.3 vs. 26.5% mortality in conservative group at day 28 (95% CI −4.8 to 20.6) –5 mesenteric ischemic events in conservative group
HOT-ICU (4)	RCT in patients with acute hypoxemic respiratory failure	PaO_2_ 60 mmHg (Only had measured SaO_2_ corresponding, median ranged from 91 to 94%) [1,441]		PaO_2_ 90 mmHg (Only had measured SaO_2_ corresponding, median ranged from 94 to 98%) [1,447]		–No significant difference in 90 day mortality –No significant between-group different in days alive without life support –Similar occurrence of organ injury
Semler et al. (11)	Pragmatic, cluster-randomized, cluster-crossover trial in mechanically ventilated adults	Low SpO_2_ 90%; goal range, 88–92% [808]	Intermediate SpO_2_ 94%; goal range, 92–96% [859]		High SpO_2_ 98%; goal range, 96–100% [874]	–No difference in ventilator-free days or 28-day mortality between all three groups.
Tyagi et al. (46)	Single center cohort in mechanically ventilated patients	Evaluated dose and duration of PaO_2_ associated with mortality				–PaO_2_ positively correlated with mortality above 200 mmHg and below 100 mmHg
**Studies of patients with critical illness**						
Oxygen-ICU (7)	RCT in ICU patients with anticipated stay > 72 h	PaO_2_ 70–100 mmHg (SpO_2_ 94–98%) [218]		PaO_2_ up to 150 mmHg (SpO_2_ 97–100%) [216]		–Terminated early due to difficulty enrolling Conservative group had lower mortality (11.6 vs. 20.2%, *p* = 0.01)
HyperS2S (8)	2 × 2 factorial RCT for patients with septic shock (evaluated hypertonic saline and hyperoxia)	Target SpO_2_ of 88–95% [223]		Hyperoxia with FiO_2_ at 1.0 [219]		–Trial stopped prematurely for safety reasons No significant difference in 28-day mortality (35% normoxia vs. 43% hyperoxia, *p* = 0.12) Fewer adverse events in normoxia group (76 vs. 85%, *p* = 0.02), as well as fewer ICU weakness and atelectasis
Gelissen et al. (6)	RCT in critically ill patients with SIRS	Target PaO_2_ 8–12 kPa SpO_2_ Median: 95.8 (94.6–97) [205]		Target PaO_2_ 14–18 kPa SpO_2_ Median: 97.2 (95.6–98.5) [195]		–No significant difference in duration of ventilation, in-hospital mortality, AKI, or MI –Not powered for smaller effect size
Schmidt et al. (3)	2 × 2 factorial RCT in comatose adults with out-of-hospital cardiac arrest (evaluated blood pressure targets as well)	PaO_2_ 9–10 kPa (68–75 mm Hg) [394]		PaO_2_ 13–14 kPa (98–105 mm Hg) [395]		–Similar incidence of death or severe disability or coma between both groups of oxygenation targets.

RCT, randomized controlled trial; SIRS, Systemic Inflammatory Response Syndrome.

#### SpO_2_ goals in patients with acute hypoxemic respiratory failure or mechanically ventilated

In patients with acute hypoxemic respiratory failure, ARDS, and patients receiving mechanical ventilation, multiple recent studies have found no difference in outcomes between liberal and conservative oxygen targets (2, 4, 5). Semler et al. assessed outcomes in mechanically ventilated patients with three SpO_2_ target ranges [low (88–92%), intermediate (92–96%), or high (96–100%)], and found no significant difference in ventilator-free days or mortality between the three target groups (11).

Only one study of patients with respiratory failure, the LOCO_2_ study, suggested possible harm to lower oxygenation targets, in patients with ARDS. It compared conservative (PaO_2_ 55–70 mmHg or SpO_2_ 88–92%) and liberal (PaO_2_ 90–105 mmHg or SpO_2_ ≥96%) oxygenation goals, and while the study was stopped early due to low likelihood of significant difference, there were 5 mesenteric ischemic events present in the conservative oxygenation group (10).

Tyagi et al. conducted a recent retrospective cohort study analyzing the relationship between the occurrence of hyperoxia with mortality in mechanically ventilated patients. They identified a U-shaped curve, where PaO_2_ was positively correlated with mortality below 100 mmHg and above 200 mmHg. This study found that exposure to severe hyperoxia, defined as PaO_2_ >200 mmHg, correlated with higher mortality (OR 1.29; 95% CI 1.04–1.59) (46).

A systematic review and meta-analysis by Cumpstey et al. included eight trials of mechanically ventilated patients (47). They found a possible increased long-term mortality with targeting hyperoxia versus normoxia, and no difference in outcomes with targeting relative hypoxemia versus normoxemia.

#### SpO_2_ goals in critically ill patients

As noted, studies have also been done in critically ill patients (not necessarily with acute hypoxemia or ventilated), largely focused on the question of benefits or harms with hyperoxia, and also comparing different target ranges for SpO_2_. These have generally confirmed either harm or neutrality with higher oxygen targets.

The HyperS2S study, which was a two-by-two factorial design study including an evaluation of normoxia versus hyperoxia in patients with septic shock, used the same oxygenation target of 88–95% in the normoxia group as the ARDS studies mentioned above. Compared to hyperoxia (using an FiO_2_ of 1.0 regardless of SpO_2_), the normoxia group had fewer adverse events and no significant difference in 28-day mortality (35% in normoxia vs. 43% in hyperoxia, *p* = 0.12) (8). The Oxygen-ICU trial in ICU patients with expected stay >72 h, was terminated early due to difficulty enrolling patients, but the study showed lower mortality in the conservative oxygenation group (SpO_2_ 94–98%) compared to the “conventional” oxygenation group (SpO_2_ 97–100%) (11.6% vs. 20.2%, *p* = 0.01) (7).

Gelissen et al. compared “low-normal” to “high-normal” oxygenation targets in ICU patients who met two or more criteria for the Systemic Inflammatory Response Syndrome (SIRS) (6). They found no significant different in organ dysfunction between the arms, though the study may have been underpowered to find a difference. Schmidt et al. examined restrictive and liberal oxygenation targets in comatose adults after out-of-hospital cardiac arrest, with a lower target in the liberal arm than Gelissen et al. (3). In this population of critically ill adults, they found no difference in death or severe disability or coma between the two arms.

The Improving Oxygen Therapy in Acute-Illness (IOTA) systematic review, a meta-analysis of 25 randomized controlled trials (RCTs) comparing liberal and conservative oxygen therapy in acutely ill adults, evaluated liberal and conservative oxygen strategies defined either by an FiO_2_ or target SpO_2_ value (9). This analysis found that a 1% increase in SpO_2_ was associated with a 25% increase in relative risk of in-hospital mortality. Overall, liberal oxygen strategies carried an increased relative risk for in-hospital mortality (1.21, 95% CI 1.03–1.43), 30-day mortality (1.14, 95% CI 1.01–1.29), and mortality at longest follow-up (1.10, 95% CI 1.00–1.20).

Across the several ranges of oxygenation targets and several populations examined, it appears that low-normal SpO_2_ targets and high-normal SpO_2_ targets result in similar outcomes. Supra-normal target ranges (hyperoxia) may be harmful.

### Context-specific considerations in LMICs

#### Bias in pulse oximetry measurements

Pulse oximetry (SpO_2_) does not perfectly correlate with arterial oxygen saturation (SaO_2_) as measured by blood gas, and prior studies have found multiple potential sources of inaccuracy, particularly as patients become sicker, as denoted by rising lactate and hypoxia (48, 49). These include severe hypoxemia, low perfusion, patient movement, and severe anemia. The quality and appropriate use of the device also play a significant role (50).

The accuracy of pulse oximetry can also be influenced by skin pigmentation, leading to measurement bias. Studies have demonstrated discrepancies in oxygen saturation detected by pulse oximetry and true arterial oxygen saturation based on patients’ skin tone (51). Valbuena et al. evaluated discrepancies by race between pulse oximetry and arterial oxygen saturation among patients in medical and surgical wards. They found that, compared to white patients, Black patients had higher odds of having occult hypoxemia noted on arterial blood gas that was not detected by pulse oximetry, with occult hypoxemia defined as arterial blood oxygen saturation (SaO_2_) of <88% despite a pulse oximetry (SpO_2_) reading of ≥92% (52). Henry et al. found that, compared to white patients, Black, Asian, and American-Indian patients admitted to the ICU or undergoing surgery during hospitalization were more likely to experience occult hypoxemia; however, the differences were only significant for Black patients after adjustment (OR 1.65; 1.28–2.14; *p* < 0.001) (53). A systematic review and meta-analysis investigating this bias in pulse oximetry measurement was performed by Shi et al., and found that compared to standard SaO_2_ measurement, pulse oximetry overestimates oxygen saturation in people with higher levels of skin pigmentation (pooled mean bias 1.11%; 95% CI 0.29–1.93%) and people described as Black/African American (1.52%; 0.95–2.09%) (54).

Henry et al. also looked at clinical outcomes of these disparities, and found that occult hypoxemia was associated with increased odds of mortality in surgical (OR 2.96; 1.20–7.28; *p* = 0.019) and ICU patients (OR 1.36; 1.03–1.80; *p* = 0.033). Occult hypoxemia was associated with fewer hospital-free days in surgical patients (−2.5 days; −3.9 to −1.2 days; *p* < 0.001) but not ICU patients (0.4 days; −0.7 to 1.4 days; *p* = 0.500) (53). This is in agreement with other studies that have found that patients with occult hypoxemia defined in this case as SpO_2_ ≥88% despite SaO_2_ <88%, were at higher risk of organ dysfunction and mortality (55).

This bias in measurement can also influence delivery of medical interventions. Gottlieb et al. investigated how these discrepancies in pulse oximetry measurements translate into racial and ethnic disparities in supplemental oxygen administration. This study found that patients of Asian, Black, and Hispanic race and ethnicity were all associated with a higher SpO_2_ for a given hemoglobin oxygen saturation. Furthermore, Asian (coefficient, −0.291; 95% CI, −0.546 to −0.035; *p* = 0.03), Black (coefficient, −0.294; 95% CI, −0.460 to −0.128; *p* = 0.001) and Hispanic (coefficient, −0.242; 95% CI, −0.463 to −0.020; *p* = 0.03) race and ethnicity were associated with lower average oxygen delivery rates (56). When controlling for the discrepancy between average SpO_2_ and average hemoglobin oxygen saturation, race and ethnicity were not associated with oxygen delivery rate. In other words, Asian, Black, and Hispanic patients received less supplemental oxygen than white patients, and this bias was explained by differences in pulse oximeter performance. Fawzy et al. similarly found that inaccuracy of pulse oximetry by race and ethnicity was associated with significantly delayed or unrecognized eligibility for COVID-19 therapies among Black and Hispanic patients (57).

The studies above demonstrate that not only is there significant bias in measurement of oxygen saturation in individuals with darker skin tones, it also translates into poorer healthcare delivery and clinical outcomes for these patients, which further perpetuates health inequities. The literature also highlights that the magnitude of inaccuracy with different skin tones is variable and not well-quantified, differing by device and other patient factors, including low perfusion (49, 58). These studies were all performed in HICs, and there is a critical paucity of such data in LMICs. In our LMIC study settings of Kenya, Malawi, and Rwanda, most patients are Black. In addition, the inability to monitor oxygen saturation continuously or frequently in these settings also increases the risk of late identification of hypoxemia.

#### Oxygen conservation

While the optimal SpO_2_ target range is not known, we do know that high levels of SpO_2_ can be harmful for patients (46). In addition, the lack of adequate supplemental oxygen supply is still a significant barrier to the provision of adequate oxygen therapy in many LMICs (25). Geographical constraints to oxygen delivery and periods of high demand, among other logistical barriers, highlight the insufficient access to oxygen in many health systems across the world (59, 60). This is confirmed by World Health Organization (WHO) data that indicated that 35% of LMIC hospitals evaluated did not have any access to supplemental oxygen (61). While HIC settings generally do not need to consider the possibility that higher SpO_2_ target ranges could result in shortages of oxygen and therefore critical hypoxemia for a proportion of hypoxemic patients, this is a relevant consideration for LMIC settings. If unnecessarily high SpO_2_ targets result in an inability to provide oxygen to a proportion of hypoxemic patients, then this will result in worse outcomes overall for the population of hypoxemic patients. The risks of hyperoxia, coupled with the fact that supplemental oxygen is a scarce resource that can be depleted in LMIC settings, highlights the need to consider the full context and population consequences when deciding on an SpO_2_ target range. Prior studies have suggested that even small differences in SpO_2_ target ranges can have profound impacts on oxygen consumption (62). Semler et al. also demonstrated that lower SpO_2_ targets were associated with overall lower FiO_2_ despite no difference in hypoxemia (SpO_2_ <85%) (11). This demonstrates a lower SpO_2_ target could decrease overall oxygen resource utilization without carrying a risk for worse hypoxemia.

#### Lack of consistent access to arterial blood gases

Many LMIC sites do not have consistent access to arterial blood gases. This means that pulse oximetry is crucial for the ability to recognize and treat hypoxemia, even recognizing the limitations of pulse oximetry noted above (24, 63).

The lack of consistent access to arterial blood gases also means that LMIC sites will often not be able to identify the subpopulation of hypoxemic patients who also have hypercapnia. A chosen SpO_2_ target range must be safe for the subpopulation of hypoxemic patients who are also hypercapnic. Higher SpO_2_ targets in this population can dangerously worsen hypercapnia due to an increase in dead space, the Haldane effect, and a decrease in minute ventilation (64). For this population, as noted above, the accepted target range is 88–92% (35–41).

#### Altitude

The impact of altitude on median SpO_2_ in a healthy population is not specific to LMIC settings, but is another contextual factor that could impact the choice of SpO_2_ target range (26–28). Median SpO_2_ values decrease in healthy populations as altitude increases (27, 28). Four of our five study sites are located at an altitude above 1,500 m, and thus likely have a somewhat lower median SpO_2_ value for their healthy populations than sea-level locations. While it is not well-understood whether or how SpO2 targets should be adjusted based on the “normal” values for a population at a given altitude (26), sites at higher altitudes may want to choose lower SpO2 target ranges to account for the lower “normoxia” ranges of their own population.

### SpO_2_ target range of 90–94%

Based on the review of available evidence and discussion among investigators and clinicians across study sites, we preliminarily decided to use 90–94% as the target SpO_2_ range for our trial of high flow versus standard flow oxygen (Table 4). This was consistent with precedent from prior trials, multiple society guidelines, and the evidence we have for liberal versus conservative targets. Our choice of target SpO_2_ range of 90–94% also accounts for contextual factors, including pulse oximetry bias in patients with dark skin pigment, oxygen conservation goals, the need to consider hypercapnic patients, and the impact of altitude on the median SpO_2_ of a population. Recognizing that pulse oximeters have a range of performance characteristics, (65, 66) we are mitigating the risk of occult hypoxemia by using pulse oximeters that have expanded evidence for accurate performance with a range of skin pigmentation levels (67); locations with less-studied or poorly-performing pulse oximeters may want to choose a higher range.

**TABLE 4:** Advantages and disadvantages of an 90-94% target SpO_2_ in Kenya, Malawi, and Rwanda.

Advantages	Disadvantages
• Prior ARDS trials have previously set SpO_2_ targets in a similar range • This range accounts for patients at risk of hypercapnia, as well as prevents potential harm from hyperoxia • Prior society and expert guidelines differ, but most recommendations include components of this range • This range encourages conservation of oxygen resources by avoiding unnecessary hyperoxia in some patients that could result in inadequate oxygen resources for other patients • Providing an upper bound avoids the risks of hyperoxia	• Patients with darker skin tones are at risk of occult hypoxemia due to differential accuracy of pulse oximeters by skin tone • Hidden hypoxemia is associated with delayed clinical care and poorer outcomes • Sicker patients with poor perfusion may have inaccurate pulse oximeter measurements • This range does not fully take into account adjustments to SpO_2_ target range for patients at risk of hypercapnia • The range is relatively narrow, and this target range may be difficult to achieve given the dynamic nature of SpO_2_ in some patients over time

## Conclusion

The optimal SpO_2_ target range for acute hypoxemic adults has not been established by trial evidence. Landmark studies of hypoxemic respiratory failure have often used a range of 88–95%, and society guidelines recommend at least an SpO_2_ > 90% (88–92% for patients with hypercapnia). A lower SpO_2_ target might lead to occult hypoxemia and worse outcomes in patients with darker skin pigmentation or for patients in settings where monitoring may be limited. However, risks of hyperoxia and depletion of oxygen resources must be considered when recommending a target oxygenation goal. Inability to identify hypercapnic patients and the impact of altitude are also important considerations. Based on available evidence from HICs of outcomes between liberal and conservative oxygen targets, we preliminarily chose an SpO_2_ target of 90–94%, determined by an accurate pulse oximeter, as a reasonable SpO_2_ range that might mitigate risks from hyperoxia while avoiding hypoxemia and conserving oxygen in our study settings.

We used precedent from prior trials, society guideline recommendations, direct evidence from HIC trials, and the input of content and context experts in LMICs to determine a reasonable target SpO_2_ range for our LMIC contexts. Nonetheless, our work points to the need for ongoing critical care research in LMICs, to create robust evidence that arises from and is applicable to LMIC settings. Improving equity in evidence must include prioritization of research in diverse settings globally.

## Author contributions

ER and TT conceived the review. AH and ER conducted the initial review and synthesis of data. AH and SG drafted the initial manuscript. All authors participated in discussions of the literature and contextual factors, reviewed and interpreted the studies included in the manuscript, and contributed toward the preparation of this manuscript.
